# Limited genetic changes observed during *in situ* and *ex situ* conservation in Nordic populations of red clover (*Trifolium pratense*)

**DOI:** 10.3389/fpls.2023.1233838

**Published:** 2023-08-09

**Authors:** Jenny Hagenblad, Karolina Aloisi, Petter Marum, Linda Öhlund, Svein Øivind Solberg, Åsmund Asdal, Anna Palmé

**Affiliations:** ^1^ Department of Physics, Chemistry and Biology, Linköping University, Linköping, Sweden; ^2^ Nordic Genetic Resource Center (NordGen), Alnarp, Sweden; ^3^ Malmö University, Malmö, Sweden; ^4^ Graminor AS, Ridabu, Norway; ^5^ Plant Breeding, Lantmännen, Svalöv, Sweden; ^6^ Department of Agricultural Sciences, Faculty of Applied Ecology, Agricultural Sciences and Biotechnology, Inland Norway University of Applied Sciences, Elverum, Norway

**Keywords:** *Trifolium pratense*, *in situ* conservation, *ex situ* conservation, genetic diversity, gene bank

## Abstract

**Introduction:**

*In situ* and *ex situ* conservation are the two main approaches for preserving genetic diversity. The advantages and disadvantages of the two approaches have been discussed but their genetic effects have not been fully evaluated.

**Methods:**

In this study we investigate the effects of the two conservation approaches on genetic diversity in red clover. Seed samples collected from wild populations in Sweden and Norway in 1980, their subsequent generations created during seed regeneration at the gene bank and samples recollected from the same location as the original samples, were analyzed with microsatellite markers, alongside reference samples from cultivars.

**Results:**

Overall, there was a differentiation between cultivars and the wild material and between wild material from Sweden and Norway. In general, the original collections clustered together with the later generations of the same accession in the gene bank, and with the recollected samples from the same location, and the level of diversity remained the same among samples of the same accession. Limited gene flow from cultivated varieties to the wild populations was detected; however, some wild individuals are likely to be escapees or affected by gene flow.

**Discussion:**

In conclusion, there were examples of genetic changes within individual accessions both *in situ* and *ex situ*, as is also to be expected in any living population. However, we observed only limited genetic changes in both *in situ* and *ex situ* conservation over the generations included in this study and with the relatively large populations used in the *ex situ* conservation in the gene bank at NordGen.

## 1 Introduction

Gene banks across the world serve the dual purpose of conserving threatened biodiversity and making their genetic resources accessible to plant breeders, researchers and others. With these goals in mind, national, regional and global efforts have collected genetic resources of a wide range of species, which for plants includes both crop landraces and cultivars as well as accessions of their wild relatives (e.g. Loskutov, 1999; Upadhyaya et al., 2008; Asdal et al., 2019). The aim has been to capture as much relevant genetic diversity as possible. As a result, some 7.4 million plant accessions are being conserved *ex situ*, the majority in more than 1750 seed gene banks worldwide (FAO, 2010).

Despite this impressive effort, concerns have been raised both regarding gaps in the collections and about the quality of long-term conservation in gene banks (FAO, 2010; Engels and Ebert, 2021). Part of the latter problem is the genetic effects of *ex situ* conservation in gene banks (e.g. Fu, 2017). Population genetic theory predicts that genetic drift during propagation will lead to loss of genetic diversity. In addition, gene flow (through contamination or pollen transfer), selection and mutation may, to a larger or smaller extent, change an *ex situ* preserved population and potentially adapt it to the gene bank environment. Empirical studies have confirmed that cross-pollinating species do change genetically during gene bank conservation (e.g. Chebotar et al., 2003; van Hintum et al., 2007; Solberg et al., 2015) and have detected the presence of genetic drift (Parzies et al., 2000; Chebotar et al., 2002; Chebotar et al., 2003). The extent of change can be minimized by appropriate gene bank management approaches, but these are often restricted by limited budgets, facilities, and personnel (FAO, 2010).

An alternative to gene bank conservation is preservation of wild populations *in situ*, in their natural habitat. While threatened by the risk of extinction, e.g. from environmental changes, habitat loss or changes in land use, these populations also have the potential to adapt to the changing environment (Exposito-Alonso et al., 2019; Fu et al., 2019), thus improving their chances of long-term survival. However, changes in natural populations can also be a threat, leading to reduced adaptation and/or loss of unique properties. Especially small populations can be problematic and display limited adaptation to local conditions (Leimu and Fischer, 2008), most likely due to random genetic drift (Blanquart et al., 2012). Another threat to *in situ*-conserved populations can be gene flow from cultivated plants. This occurs in most crops when cultivated next to closely related wild species (Ellstrand et al., 1999; Uwimana et al., 2012) and can result in maladaptive changes or even extinction (Todesco et al., 2016).

Red clover (*Trifolium pratense* L.) is an important component of fodder production around the world and is widely cultivated in the Nordic countries (Denmark, Norway, Sweden, Finland and Iceland). It is typically grown in mixtures with grasses such as timothy (*Phleum pratense* L.) and meadow fescue (*Festuca pratensis* Huds.) (Boller et al., 2010). The ability of red clover to fix nitrogen through symbiosis with *Rhizobium* bacteria reduces the need for supplementary nitrogen fertilizers (Boller and Nösberger, 1987; Nesheim and Øyen, 1994). In addition, clover has a low fiber and high protein content, which is beneficial to fodder quality (Katoch, 2022) and it is an important source of plant protein production in Nordic countries. The species is pollinated by insects, frequently by long-tongued bumblebees (*Bombus* sp) (Goulson et al., 2005) and it is self-incompatible (Williams and Silow, 1933). In cultivation, red clover is a fairly short-lived perennial, which can cause problems for farmers who may need to reseed their leys as frequently as every third year (Micke and Parsons, 2023). Studies have shown that red clover populations can quickly adapt to the climate in which they have been cultivated for generations (Collins et al., 2012) and plant breeding efforts are aiming for better survival in addition to high yields (Ergon and Bakken, 2022; Korelina et al., 2022).

Populations of red clover from natural environments have been an important starting point for plant breeding (Helgadóttir et al., 2000) and wild populations are expected to hold variation of significance for future crop improvement (Zanotto et al., 2021). For this reason, the collection and conservation of wild red clover have been a priority for the Nordic Gene Bank (NordGen) since its inauguration in 1979. Local populations are expected to be adapted to the climate and conditions of the area, provided that enough time has passed since establishment and that the population size is large enough (Leimu and Fischer, 2008), which seems to be the case in red clover (Joshi et al., 2001). In order to include local diversity in the Nordic *ex situ* collection, efforts have been made to sample across the whole geographic distribution in the Nordic countries. Today the Nordic collection includes over 500 red clover accessions (Asdal et al., 2019; GENBIS, 2022) of which half are wild populations collected from respectively Sweden (98 accessions), Norway (94 accessions), Finland (14 accessions), and Denmark (1 accession).

**Figure 1:**
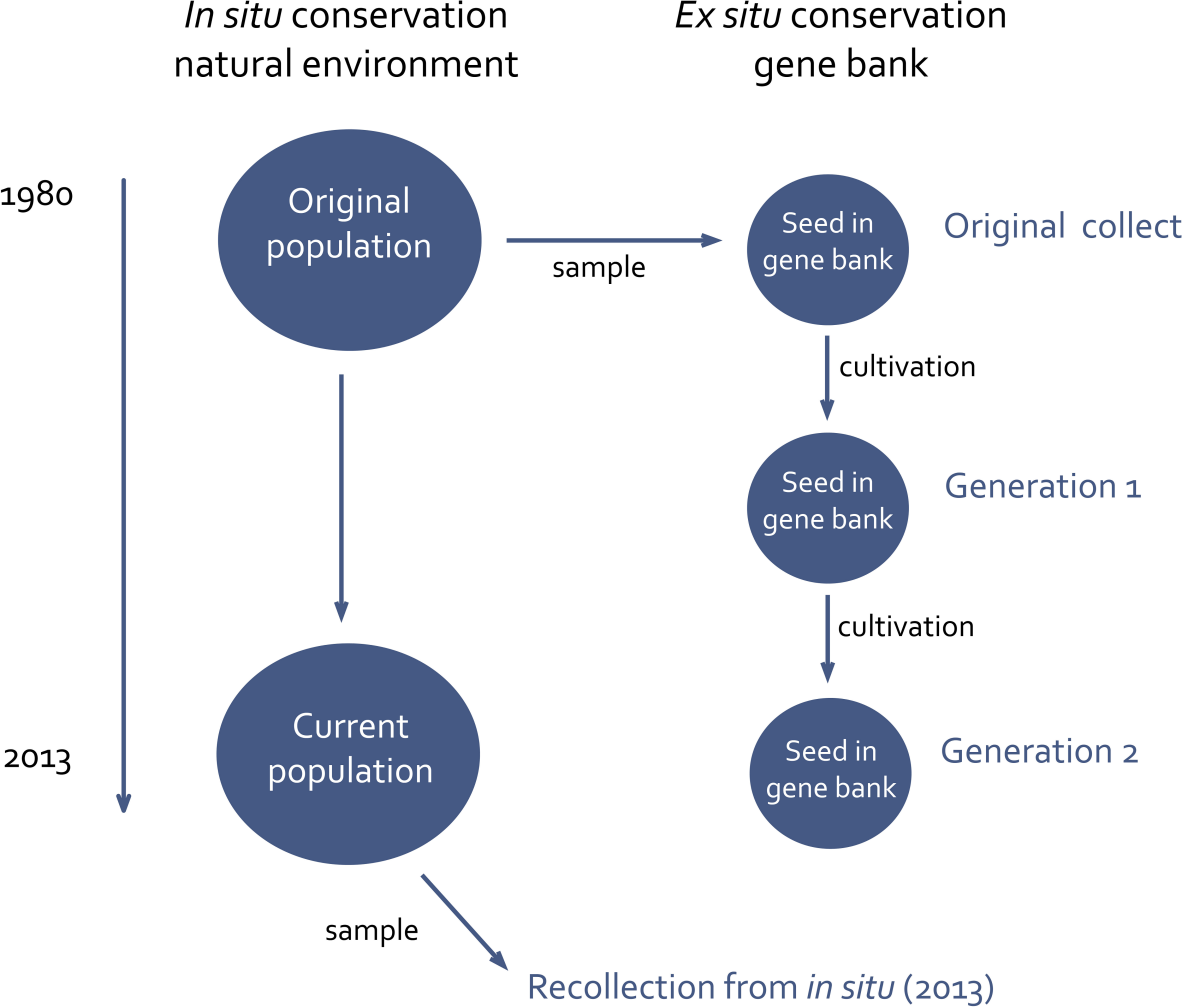
Investigation setup. Samples for molecular analysis are taken from the original collect, generation 1, generation 2 and from the wild population in 2013 (all indicated with blue text).

The main aim of this study is to investigate and compare the genetic effects of *in situ* and *ex situ* conservation using red clover collected in Sweden and Norway as an example. We examine the genetic diversity and composition of the accessions at different time points. The study includes both populations conserved in nature (*in situ*) and in the gene bank (*ex situ*) (**Figure 1**) to evaluate the evolutionary effects of gene bank propagation and processes affecting populations in nature.

## 2 Materials and methods

### 2.1 Study material

**Figure 2:**
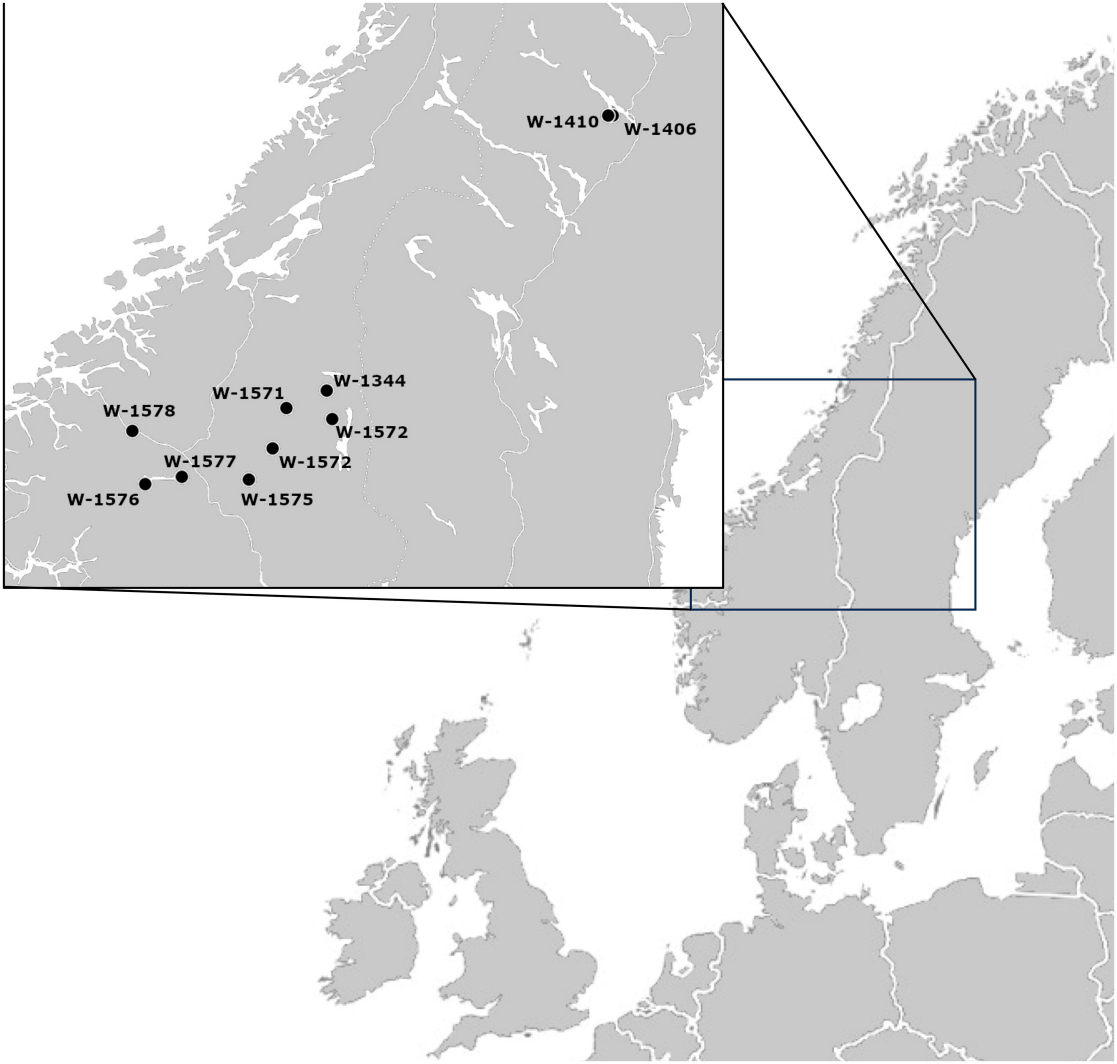
Map of the study area and the area of origin of the studied accessions.

To compare *ex situ* and *in situ* conservation of the same plant population (**Figure 1**), suitable samples were selected. As a first step, NordGen’s database was searched for red clover accessions fulfilling the following criteria: 1) collected in natural habitats, 2) sampled in the 1980s or earlier, 3) containing information on sampling location, including longitude and latitude, 4) stored at NordGen for more than one generation, and 5) with enough seeds available to conduct the study. Key issues were possibilities to compare different generations at the gene bank and adequate information to identify the original sampling location for carrying out recollections. After applying the above criteria, two geographic focus areas were selected, Västerbotten in Sweden and Innlandet in Norway (**Figure 2**). In both areas red clover accessions were sampled in 1980, but a larger number of suitable accessions were identified from Norway.

Collection trips to Sweden and Norway were conducted in the summer of 2013: Västerbotten in Sweden in June and Hedmark/Oppland in Norway in August. Leaf samples were collected and dried with silica gel in the field. For the Swedish accessions, the name of the nearby village and the longitude and latitude with four digits were available from the sampling in 1980, however, no details on the exact location had been noted. In 2013, it was possible to sample leaves from the same villages as two of the Swedish accessions sampled in 1980 (**Table 1**; **Supplementary Table S1**). In Norway the documentation was more detailed and the person originally responsible for the sampling in 1980, Petter Marum, was able to take part in the recollection. The original sampling location of nine accessions could therefore be identified and samples from eight of these are included in this study (**Table 1**; **Supplementary Table S1**).

**Table 1:** Studied accessions, their origin, and genetic diversity.

Code	Accession number	Accession name	Origin country (county)	Description	Diversity (h)	No polymorphic loci (%)	No individuals analysed
W-1406-orig	NGB 1406	REMBACKA GB0201	Sweden (Västerbotten)	Collected in grassland, no cultivation for the last 30 years. Could be semi-wild.	0.713	21 (95%)	16
W-1406-gen1				First *ex situ* regeneration, Jokioinen	0.763	21 (95%)	15
W-1406-re				Collected in field margin, modern forage cultivar mix sown in area close by	0.771	21 (95%)	16
W-1410-orig	NGB 1410	SKARPMYRBERG GB0601	Sweden (Västerbotten)	Collected in grassland, no cultivation for at least 20 years	0.656	21 (95%)	6
W-1410-gen1				First *ex situ* regeneration, Landvik, cage isolation	0.741	21 (95%)	16
W-1410-re				Collected in an abandoned field, no red clover sown for at least 40 years	0.728	21 (95%)	16
W-1571-orig	NGB 1571	TRØA 09-6-48-1	Norway (Hedmark)	Collected from grassland on a south facing slope grazed by sheep. Area next to a field.	0.690	21 (95%)	13
W-1571-gen1				First *ex situ* regeneration, Løken, distance isolation	0.704	21 (95%)	15
W-1571-gen2				Second *ex situ* regeneration, Landvik, cage isolation	0.726	20 (91%)	14
W-1571-re				Sampled from the same location as in 1980. Collected in field margin	0.739	21 (95%)	16
W-1572-orig	NGB 1572	TORRUD 09-6-48-2	Norway (Hedmark)	Collected from an old meadow not used since 1953, 100 plants	0.733	21 (95%)	16
W-1572-gen1				First *ex situ* regeneration, Løken, distance isolation	0.732	21 (95%)	11
W-1572-gen2				Second *ex situ* regeneration, Landvik, cage isolation	0.725	21 (95%)	14
W-1572-re				The original old meadow is gone, the road has been rebuilt and an access road and new buildings constructed. Samples collected on the roadside	0.742	21 (95%)	16
W-1574-orig	NGB 1574	SIKSJØLIA 09-6-48-5	Norway (Hedmark)	Collected from grazed mountain grassland on a southwest facing slope	0.645	20 (91%)	16
W-1574-gen1				First *ex situ* regeneration, Løken, distance isolation	0.653	21 (95%)	16
W-1574-re				The area where the original collection was made is now ploughed and re-sown. Collection made from the neighbouring field where no red clover has been sown.	0.690	21 (95%)	15
W-1575-orig	NGB 1575	TRØA 09-6-48-6	Norway (Hedmark)	Collected from an old meadow not used for many years (at least for 25 years)	0.724	21 (95%)	14
W-1575-gen1				First *ex situ* regeneration, Løken, distance isolation	0.683	21 (95%)	15
W-1575-gen2				Second *ex situ* regeneration, Landvik, cage isolation	0.692	21 (95%)	16
W-1575-re				Collection in the area sampled in 1980, now partly cut as a lawn. No cultivation during the last 60 years.	0.695	21 (95%)	12
W-1576-orig	NGB 1576	AUKRUST 09-6-49-1	Norway (Oppland)	Collected in a field margin close to the farm, southwest facing slope of the valley, 100 plants.	0.708	21 (95%)	15
W-1576-gen1				First *ex situ* regeneration, Løken, distance isolation	0.724	21 (95%)	16
W-1576-re				Collection in an area with sparce deciduous forest, next to a pasture which has been recently sown. The original collection in 1980 most likely took place in the margin of the latter.	0.697	21 (95%)	16
W-1577-orig	NGB 1577	Øy 09-6-49-2	Norway (Oppland)	Collected from an old abandoned dry meadow on a southeast facing slope, 100 individuals. The presence of non-native lucerne suggests that the meadow have been sown with cultivars.	0.760	21 (95%)	15
W-1577-gen1				First *ex situ* regeneration, Løken, distance isolation	0.707	21 (95%)	13
W-1577-re				Most of the area that was sampled in 1980 no longer contain red clover (cultivated land). Samples taken in a small area between two copses of trees.	0.663	20 (91%)	7
W-1578-orig	NGB 1578	EINBU 09-6-49-3	Norway (Oppland)	Collected in natural population by the roadside, 100 plants	0.728	21 (95%)	16
W-1578-gen1				First *ex situ* regeneration, Løken, distance isolation	0.723	21 (95%)	16
W-1578-re				Collection from roadside where the original collection was made.	0.726	21 (95%)	15
W-13447-orig	NGB 13447	09-6-48-3	Norway (Hedmark)	Collected in a natural pasture grazed by cattle, 50 plants	0.682	19 (86%)	16
W-13447-gen1				First *ex situ* regeneration, Landvik, cage isolation	0.680	20 (91%)	15
W-13447-re				Collected at the same location as in 1980	0.694	20 (91%)	15
C-2183	NGB 2183	‘Molstad’	Norway	Released as cultivar 1953. Diploid. Originated from a landrace cultivated on Molstad farm, which had its origin in seeds imported in the 1850s. The most cultivated variety in Norway for a long time.	0.785	21 (95%)	16
C-2745	NGB 2745	‘Björn’	Sweden	Released 1977. Diploid. Developed from the old cultivar ‘Offer’ with the aim to improve resistance to clover rot.	0.794	21 (95%)	16
C-7786	NGB 7786	‘Pradi’	Norway	Released 1981. Diploid. Selection from semi-wild/wild Norwegian material.	0.789	21 (95%)	16
C-11155	NGB 11155	‘Nordi’	Norway	Released 1989. Diploid. Selection in Molstad for clover rot (*Sclerotinia trifoliorum*) resistance.	0.782	21 (95%)	15
C-13203	NGB 13203	‘Bjursele’	Sweden	Local cultivar released in 1962, withdrawn 2006. Diploid. Extensively used in Västerbotten.	0.782	21 (95%)	16
Lea		‘Lea’	Norway	Released 2002. Diploid. Developed at Planteforsk Løken from Syn1 2x88, Bjursele and Nordi	0.778	21 (95%)	16
L-2486	NGB 2486	Bredånger	Sweden	Originally from a harvest in 1953.	0.821	21 (95%)	16
	NGB 13205	‘Betty’	Sweden	The first tetraploid cultivar for northern Sweden. Released 1992.	Not analysed, used as reference	Not analysed	Not analysed

In addition to the material collected in these natural habitats (hereafter called wild populations) and their offspring generations at the gene bank, a selection of cultivars and landraces were included (**Table 1**). These were included because they have been cultivated in the areas where the wild populations were sampled, either before 1980, or in the period 1980 – 2013, and they could therefore potentially have hybridized with local wild populations. All the cultivars included in the analysis are diploid. A tetraploid cultivar (‘Betty’) was included as a reference for a tetraploid microsatellite pattern but was not used for further comparisons.

In total, six cultivars, one landrace and ten accessions of wild red clover stored at NordGen were genotyped. For each wild accession, individuals from three or four conservation stages were included. These were the original collection (collected in 1980, henceforth referred to as “orig”) and regeneration cycle (generation) 1 (henceforth “gen1”) and sometimes 2 (henceforth “gen2”) at the gene bank, and for all accessions individuals collected in 2013 at, or close to, the original sampling location (henceforth recollections, “re”) (**Table 1**; **Supplementary Table S1**). An “accession” will hereafter refer to all individuals of all conservation stages identified by the same accession number, including recollections. A total of 16 individuals were analyzed from each conservation stage of each accession. Such a group of individuals will henceforth be referred to as a “sample”. Some of the gene bank material included in this study has also been analyzed morphologically (Solberg et al., 2015).

### 2.2 Genotyping

Dried leaf samples were powdered in a mixer mill (Merck Retsch mm 300) with a steel ball and DNA extraction carried out using the protocol described by Doyle and Doyle (1987), with the following modifications: after transferring the DNA-containing phase (approx. 450 μl) to clean tubes, 5 μl RNase (10 mg/ml) was added. Samples were then incubated at 37°C for 30 min. DNA was precipitated with 0.8 volume cold isopropanol, gently mixed and centrifuged 10 min at 13200 rpm. The supernatants were removed, and the pellets were washed in 500 μl wash buffer (76% ethanol, 0.2M sodium acetate), left at room temperature for 20 min. and centrifuged for 5 min. at 13200 rpm. They were then rinsed in a buffer (76% ethanol, 0.01 M ammonium acetate) and centrifuged at 13200 rpm for 5 min. Discarding the supernatants, the pellets were left to dry at room temperature. Pellets were re-suspended in 50 μl double distilled water. The amounts of DNA were determined using spectrophotometer analysis (BioSpectrometer, Eppendorf or QIAxpert, Qiagen).

The material was genotyped for a total of 23 microsatellite markers developed by Kölliker et al. (2006) and Sato et al. (2005) (**Supplementary Table S2**). For genotyping, PCR conditions were as follows in 20 μl reactions: 14.6 μl double distilled water, 2 μl buffer (10X), 0.4 μl dNTP (10 μm), 1 μl (10 μm) of each of forward and reverse primers, 0.12 μl Taq-polymerase (5U/μl) and 1 μl of DNA (5 ng/μl). Touchdown PCR were run at an Eppendorf Mastercycler (**Supplementary Table S3**). Fragment analyses were carried out at Uppsala Genome Centre, SciLife Lab Uppsala, Sweden.

### 2.3 Data cleaning

In total, 655 individuals from 41 samples, stemming from 18 accessions, were analyzed for the 23 loci. Initially, individuals for which more than 30% of the loci failed (too many alleles amplified or no amplification, 62 individuals) were removed. Following this, only three individuals remained of the tetraploid cultivar ‘Betty’ (NGB 13205), which were removed. Finally, one locus (RCS3620) with more than 30% failed individuals was also removed. This resulted in a data set consisting of 590 individuals from 40 samples (17 accessions) analyzed for 22 loci which was subsequently used for data analyses (**Supplementary Table S4**).

Wild red clover is diploid and so are the cultivars included in the genetic analyses, and therefore all analyses were done assuming fully diploid data. If more than two alleles were amplified for a certain locus and individual, this was treated as missing data. However, this was an infrequent occurrence in the analyzed data set occurring in less than 2% of the diploid loci.

### 2.4 Genetic data analyses

Genetic diversity was estimated as Nei’s h (Nei, 1973) using a purpose-written Perl script. The software R (R Core Team, 2022) was used for statistical testing (ANOVA, t-tests and tests for correlation) of diversity levels and level of differentiation. R was also used for calculating summary statistics describing the genetic diversity (packages *hierfstat*, *poppr*, and *popGenReport*). The *amova.result* and *amova.test* functions in the package *poppr* were used for carrying out AMOVA and for testing for significance, respectively.

Genetic structuring was investigated by calculating pairwise F_ST_ values, through PCA and with the software *STRUCTURE* (v 2.3.4) (Pritchard et al., 2000; Falush et al., 2003). Wright’s F_ST_ (Wright, 1951) was calculated between all pairs of accessions using a purpose-written Perl script. PCA of genetic data was carried out using the *prcomp* command in R treating the number of copies of each allele at each locus for the accession or individual as independent variables.

*STRUCTURE* was run using a diploid setting with a burn-in length of 20 000 iterations followed by 50 000 iterations for estimating the parameters, with 10 repeated runs at each level of predetermined clusters (K), with K ranging from 1 to 15. The software *CLUMPP* (v 1.1.2) (Jakobsson and Rosenberg, 2007) was used to compare the outcome of individual runs with the Greedy algorithm for 4 < K < 6 and with the LargeKGreedy algorithm for K ≥ 6. The number of clusters best describing the data was evaluated from the *CLUMPP* H’ values and ΔK calculated according to Evanno et al. (2005). Results were visualized using *DISTRUCT* (v 1.1) (Rosenberg, 2004).

## 3 Results

### 3.1 Potential polyploids

The analysis described in this paper was not designed to identify polyploids. However, there are indications that a few of the analyzed plants may be polyploid. In total, there were seven individuals where four or more loci amplified more than two alleles. This could be due to technical problems or suggest that these individuals are tetraploids. Of the seven, two were individuals from the original collections in 1980 the other five were from recollections in 2013. The tetraploid reference ‘Betty’ had an average of 7.4 loci per individual producing more than two alleles (in total 32,1% of the genotyped loci).

### 3.2 Genetic diversity

**Figure 3:**
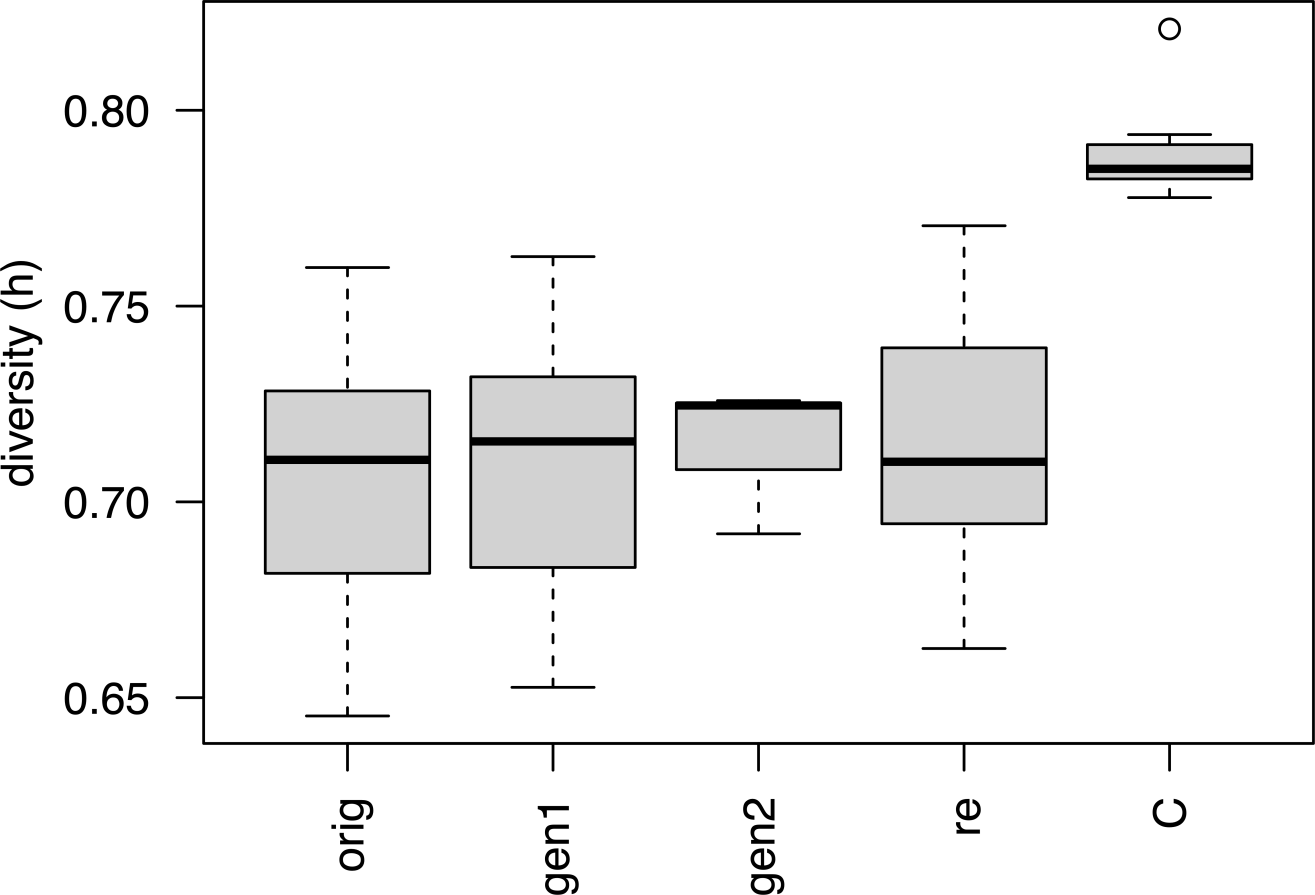
Genetic diversity for different conservation status groups. Codes for conservation status corresponds to codes used in **Table 1** with the exception of C which includes both cultivars and the landrace.

Per locus statistics are reported in **Supplementary Table S5**. Within-sample genetic diversity across all loci ranged from 0.645 for W-1574-orig to 0.821 for L-2486 (**Table 1**). When divided into groups based on the conservation stage, the “Cultivars” (including L-2486, a landrace accession) had significantly higher genetic diversity than either conservation stages (ANOVA, p = 1.44 * 10-5, **Figure 3**). Neither of the conservation stages differed significantly from each other in genetic diversity (ANOVA, p = 0.901, **Table 2**).

**Table 2:** Average genetic diversity and standard deviation for accessions with different conservation status.

Status	N samples	Average genetic diversity (h)	Standard deviation
Cultivars	7	0.790	0.013
Original collect (orig)	10	0.704	0.031
Generation 1 (gen1)	10	0.711	0.031
Generation 2 (gen2)	3	0.714	0.016
Recollection (re)	10	0.714	0.030

No significant differences in genetic diversity were detected between the original collections and the recollections of the same accession (paired t-test, p = 0.530) nor between the original collections and the first generation (paired t-test, p = 0.573), respectively. Later generations were not compared due to the limited number of populations with two or more generations. Within individual accessions there were examples both of regenerated samples having more and less genetic diversity than the corresponding original collections. Likewise, examples of both recollection samples with higher and lower genetic diversity than the original collections occurred.

### 3.3 Between-sample differentiation

To quantify the genetic differentiation among samples, F_ST_ values (Wright, 1951) were calculated between all pairs of samples (**Supplementary Table S6**). The differentiation was in general low with F_ST_ values ranging from 0.021 between W-1575-orig and W-1575-re (from Trøa in Hedmark, Norway) and 0.102 between ‘Lea’ (cultivar from Norway) and W-1574-orig (a wild/original accession collected in Siksjølia in Hedmark, Norway). Most, but not all, F_ST_ values were non-significant.

**Figure 4:**
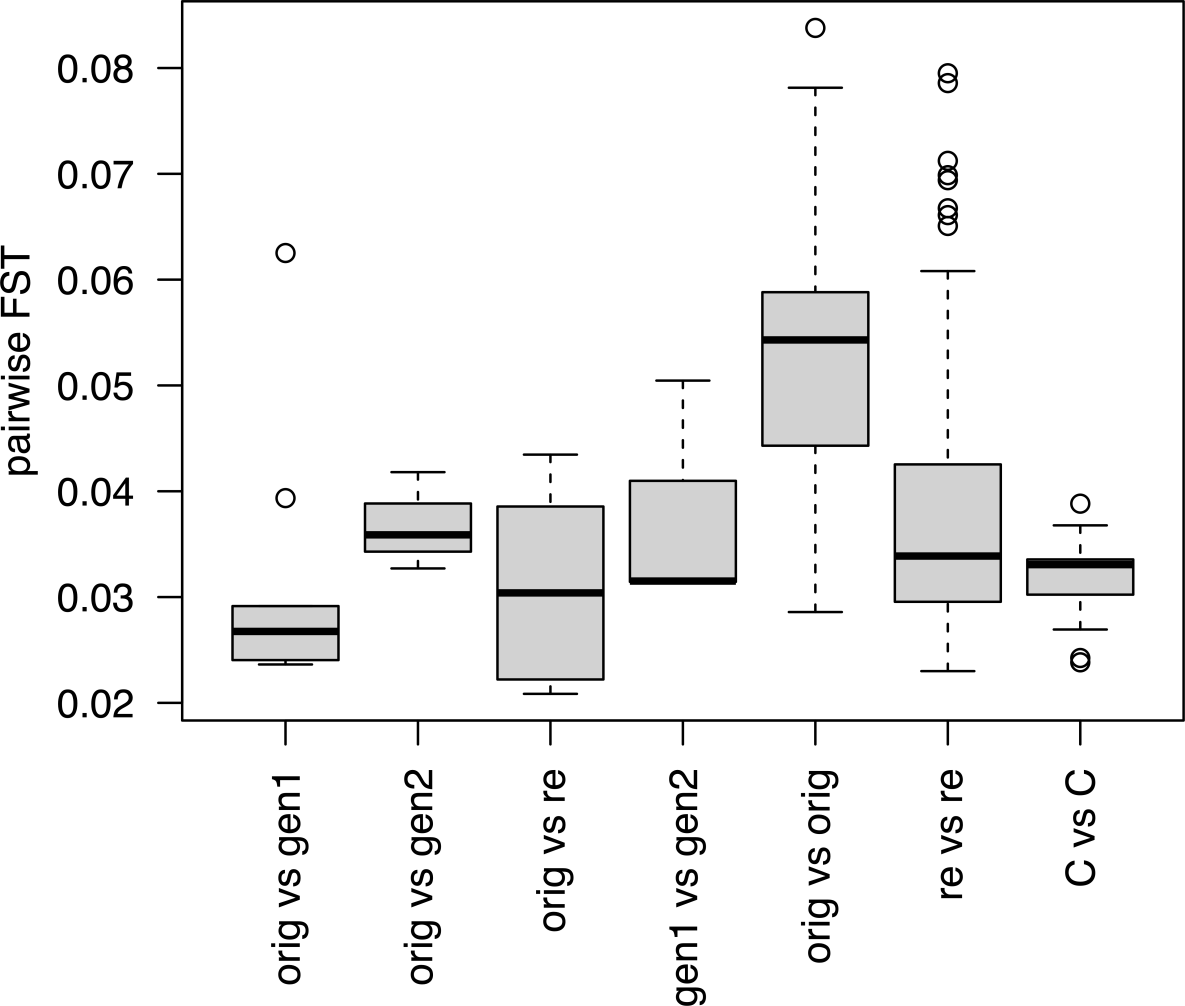
Average F_ST_ values for pairwise comparisons of different conservation statuses within an accession (first four boxes) and all pairwise comparisons within a conservation status group (last three boxes). Codes for conservation status as in **Figure 3**.

ANOVA showed that comparisons between different conservation stages (**Figure 4**) differed significantly (p < 4.68 * 10^-9^). F_ST_ values were higher for comparisons between pairs of original collections than for pairs of cultivars or pairs of recollections. Within-sample comparisons of accessions showed that F_ST_ values between the original collect and generation 1 were not significantly different from those when the original collect was compared with the recollections (paired t-test, p = 0.849). Removing accession W-1571, where the F_ST_ value between the original collect and generation 1 was unusually high (mean 0.062) did not affect these conclusions (paired t-test without W-1571, p = 0.334).

F_ST_ values calculated between the original collections and generation 2, were in two of the three available cases higher than the corresponding F_ST_ values between the original collections and generation 1 (**Supplementary Table S6**). The same was true when comparing the original collections and the recollections. W-1571 had the highest F_ST_ values when its original collect was compared with generation 1, and to a lesser extent with generation 2 or with the recollections.

On average (across all loci), 0.35 of the alleles detected in one sample were shared when compared with another sample. Samples of the same accession at different conservation stages shared an average 0.44 alleles while samples from different accessions shared significantly fewer alleles, on average 0.363 (ANOVA, p = 9.67 *10^-16^). Comparisons of the original collections of accessions with generation 1 showed a higher proportion of shared alleles (0.483) than comparisons with the recollection (0.430), though not significantly so (paired t-test, p = 0.074).

### 3.4 Genetic clustering

**Figure 5:**
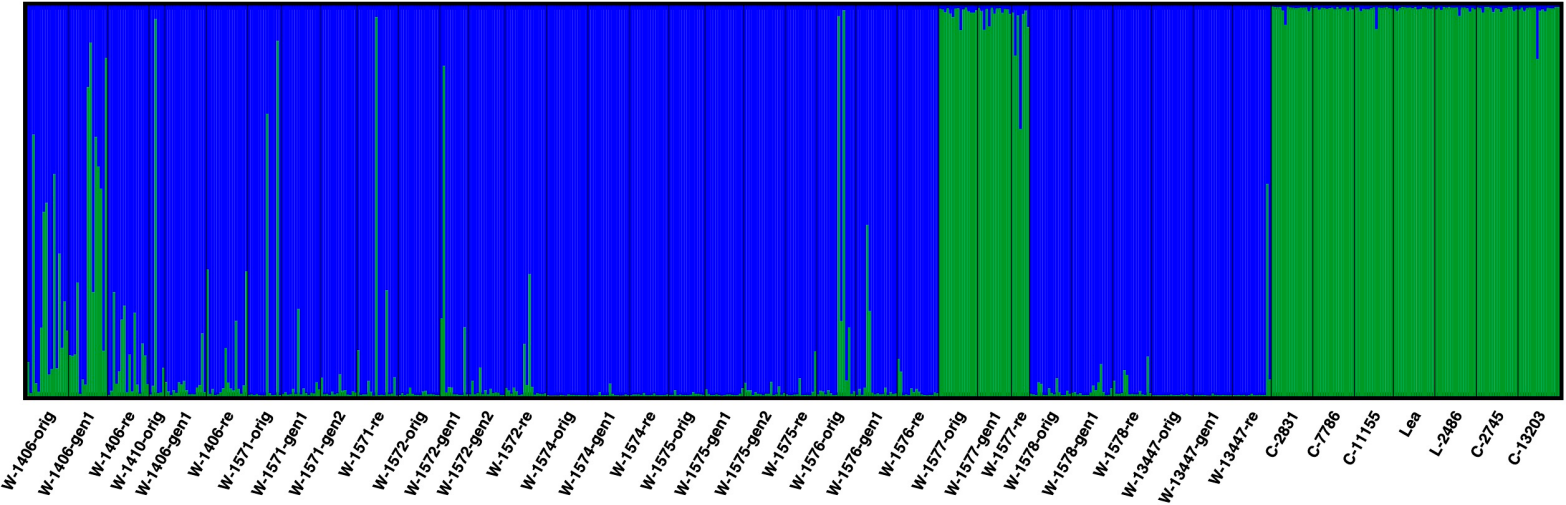
Results of *STRUCTURE* analysis of all accessions at K = 2. Each analyzed individual is represented by a vertical line and samples are separated by black vertical line. The colors of the vertical lines decode the proportion of identity of that individual to each of the clusters explained by the investigated model.

AMOVA showed that the genetic diversity primarily occurred within samples, but with significant diversity also among samples within accessions and among accessions (**Table 3**). To identify similarities among samples and accessions, the data was explored for genetic clustering using the software *STRUCTURE*. *CLUMPP* H’ and ΔK values both suggested that the full dataset was best described by two genetic clusters. At this level of clustering all cultivars, L-2486 and the samples of the accession W-1577 formed a cluster separate from all other accessions and samples (**Figure 5**).

**Table 3:** Results of AMOVA analysis of non cultivar accessions.

	Df	Sum Sq	Mean sq	Sigma	%
Between accessions	9	304.3917	33.821299	0.4683578	8.10
Between samples within accessions	23	258.1385	11.223413	0.4421846	7.64
Within samples	446	2173.3273	4.872931	4.8729311	84.26
Total	478	2735.8575	5.723551	5.7834735	100.00

**Figure 6:**
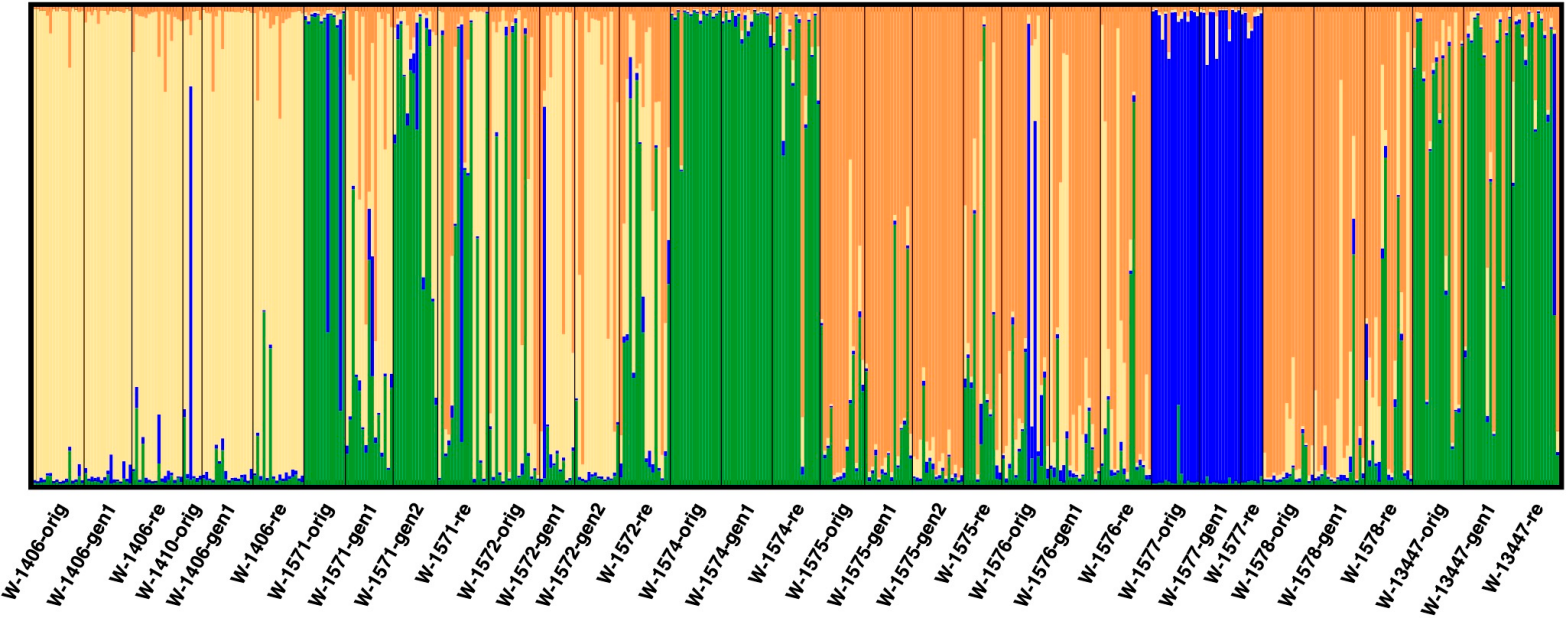
Results of *STRUCTURE* analysis excluding cultivar and landrace accessions at K = 4. Each analyzed individual is represented by a vertical line and samples are separated by black vertical line. The colors of the vertical lines decode the proportion of identity of that individual to each of the clusters explained by the investigated model.

When excluding the cultivars and landrace from the analysis (focusing only on the wild material), four clusters best described the data (**Figure 6**; **Supplementary Figure S1**). At this level of clustering, one cluster was formed by the three samples of W-1577 (blue in **Figure 6**). A second cluster (yellow in **Figure 6**) primarily consisted by the samples of W-1406 and W-1410 and to some extent W-1572. A third cluster (orange in **Figure 6**) was made up by the samples of the accessions W-1575, W-1576 and W-1578. The final cluster (green in **Figure 6**) contained the samples of the accessions W-1574 and W-13447 and to a certain extent W-1571. Generation 1 (but not 2) of W-1571, however, was primarily assigned to the yellow cluster.

**Figure 7:**
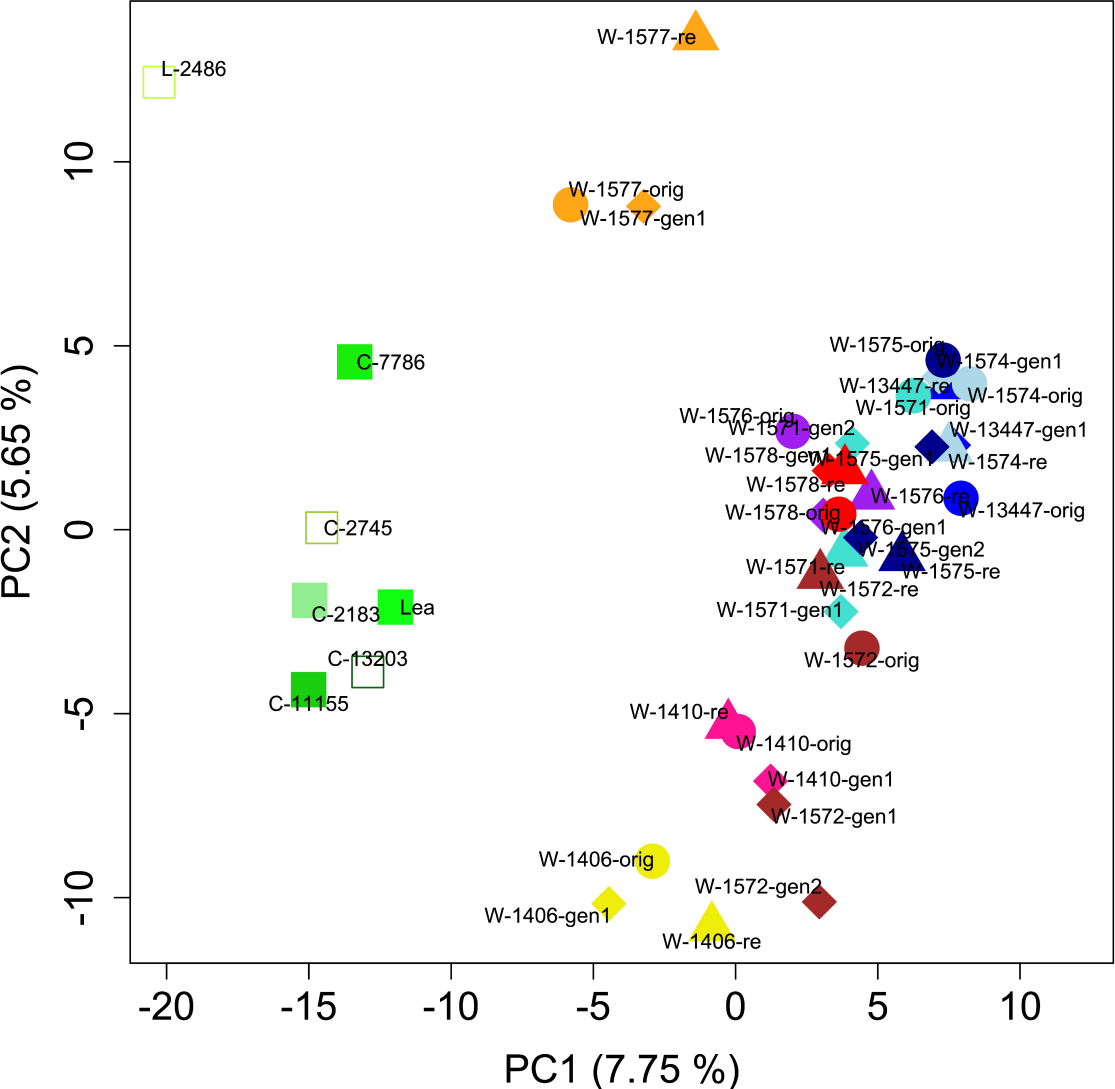
Results of PCA. Each accession is denoted by a different color. Original collections are represented by circles, regeneration samples by diamonds, recollections by triangles and cultivars and the landrace are represented by square. Open squares denote Swedish cultivars and filled squares denotes Norwegian cultivars.

**Figure 8:**
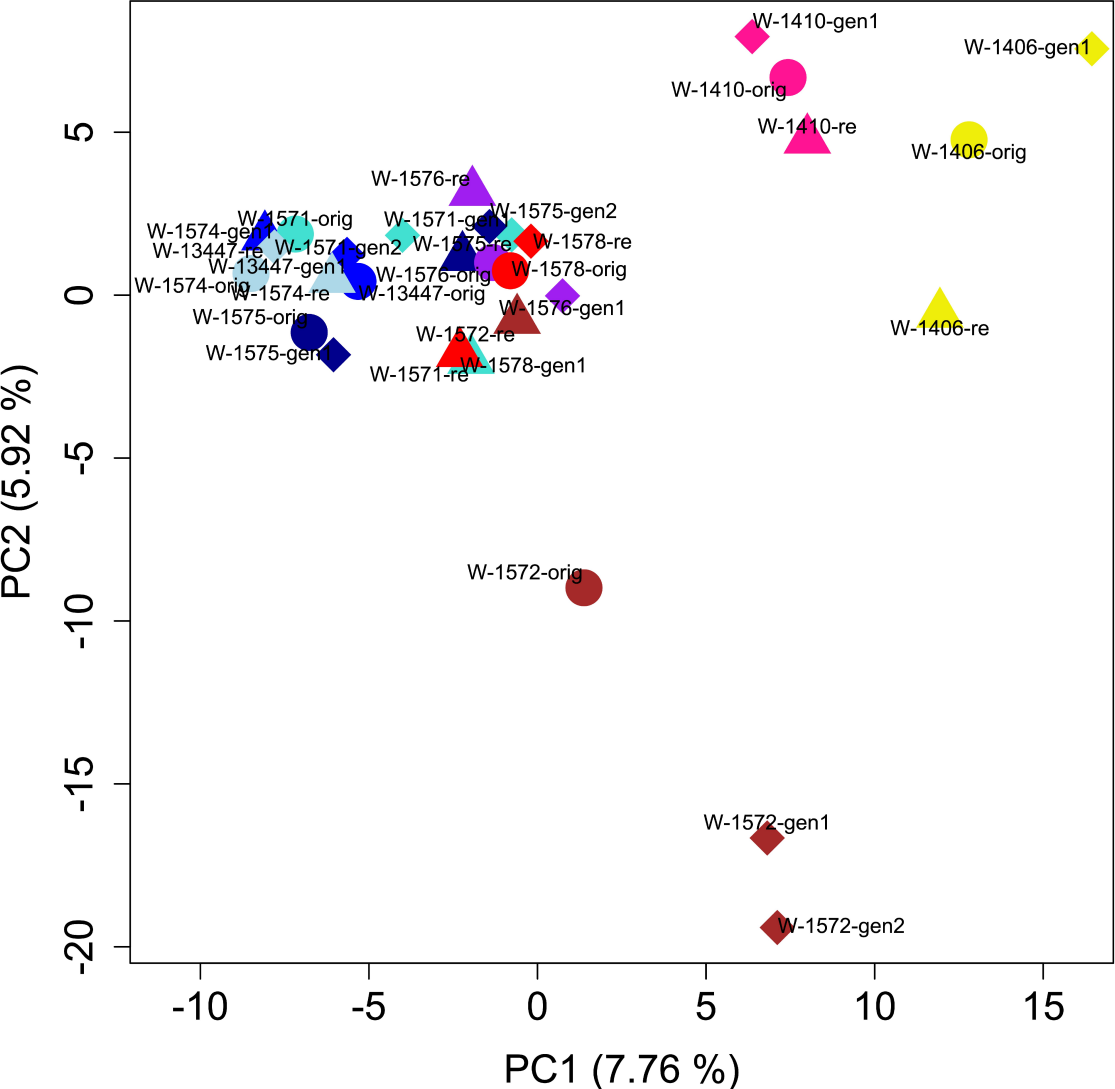
Results of PCA after excluding cultivars, the landrace and the accession W-1577. Colors and shapes as in **Figure 7**.

PCA of the full dataset further elucidated the distribution of genetic diversity. The first and second principal component (PC) explained 7.75 and 5.65% of the variation, respectively and together separated the cultivars, the landrace and the accession W-1577, thereby replicating the results of the *STRUCTURE* analysis of the full data set at K = 2 (**Figures 5**, **7**). Notably, although clustering with the cultivars in the *STRUCTURE* analysis, both L-2486 and W-1577 were differentiated from the cultivars along both PC1 and PC2, though in different directions along PC1 (**Figure 7**). When excluding cultivars, L-2486 and W-1577 PC1 and PC2 separated the Swedish accessions (W-1410 and W-1406) from the Norwegian ones with a partial overlap of W-1572 along PC1 (**Figure 8**). Pairwise F_ST_-values between original wild populations were significantly positively correlated with the geographic distances between populations (c = 0.400, p < 0.01).

For some accessions, for example W-1410 (pink in **Figure 7**), W-1578 (red), W-1574 (light blue) and W-13447 (medium blue), all samples clustered closely in the PCA. For other populations PCA results indicated a change in the genetic composition in either the regenerated samples (e.g. W-1572, brown), the recollected sample (W-1577, orange) or both (e.g. W-1575, dark blue; W-1571, turquoise). Individual level PCA showed that individuals from some samples clearly clustered with other accessions than that of designated to be their source (**Supplementary Figure S2**). However, non-cultivar individuals rarely clustered among cultivar individuals.

## 4 Discussion

Red clover is, and has been, an important fodder crop in both Sweden and Norway. Already around 1850, a considerable number of landraces of red clover existed in the Nordic countries, most of them in Sweden but also in Norway and Finland. When modern plant breeding started in the Nordic countries these landraces were the main gene pool from which the new cultivars were developed. In Sweden, modern breeding of red clover was initiated during the first half of the 20th century (Olsson, 1997). The breeding was based on genetic material collected from around the country (Julèn, 1997), and selection was based on performance with emphasize on yield but also winter hardiness and root rot resistance (e.g. Lundin and Jönsson, 1974). Later, exotic material from other European countries was included in the breeding programs to broaden the genetic base. A similar history is found in Norway where red clover breeding started some years later (Marum, 2009) but where several Swedish varieties have been marketed and exchange of genetic material has occurred.

### 4.1 Gene flow from cultivars into wild populations

Many studies support the existence of gene flow or introgression between crops and their close wild relatives (Ellstrand et al., 1999) and it is clear that alleles from cultivars can persist for many years in wild populations (Snow et al., 2010). Such gene flow can be a problem both from the view of the wild population, which can be affected by genetic swamping and/or outbreeding depression (Edmands, 2007; Todesco et al., 2016), and from an agricultural perspective if traits that increase weediness are transferred (Merotto et al., 2016; Le Corre et al., 2020). Knowledge of geneflow is therefore an important part in understanding the evolution and potential threats to wild and cultivated populations.

With the exception of NGB 7786 (‘Pradi’), the cultivars included in this study have been frequently cultivated in the investigated regions from the early 1950s and forward (**Table 1**), thus providing opportunity for hybridization. However, the genetic comparison of the cultivated and wild material supports a scenario with limited gene flow between the local cultivars and the wild red clover populations. The two types of material form separate groups/clusters (**Figures 6**, **7**, except for W-1577) and F_ST_ values are also overall higher when comparing cultivated and wild material than when comparing samples within the two groups. This pattern of differentiation between the two groups can also be seen for morphological traits (Solberg et al., 2015) and adds to the general pattern observed in red clover with differentiation between cultivated materials and most samples from the wild, for example in the Nordic region (Osterman et al., 2021; Zanotto et al., 2021) and Russia (Semerikov et al., 2002). Taken together, this suggest that most wild red clover populations are not swamped by genetic material from cultivars and that the cultivars are not heavily affected by gene flow from wild populations.

However, not all accessions and individuals conform to the general pattern of differentiation between the cultivated and wild materials, and exceptions have been observed in other studies in red clover (Semerikov et al., 2002; Osterman et al., 2021). In the *STRUCTURE* analysis, W-1577 clusters with the cultivars and landrace (**Figure 5**), which could indicate that this population is a naturalized cultivar. However, in the PCA analysis (**Figure 6**), it is intermediate between the cultivars and the Norwegian wild accessions along PC1, instead suggesting gene flow from cultivars. Geneflow is further supported by the individual based PCA (**Supplementary Figure S2**) and the fact that W-1577 has the highest diversity among the original samples (**Table 1**). A putative geneflow event must thus have occurred prior to 1980 when the original collections were made.

Among the wild accessions, there are also several individuals that could potentially be escapees from cultivation or the result of gene flow from cultivars (individuals with large portion of green in **Figure 5**; **Supplementary Figure S2**). In most cases such putative escapees occur in the original samples or regeneration samples. Only in a single case, W-1571, is a putative escapee detected in a recollection sample, suggesting that the *in situ* conserved populations have not been subjected to substantial geneflow from cultivars during the time period 1980 – 2013.

### 4.2 Genetic structure within the main groups

This study was not designed to investigate genetic structure within cultivated material, and no differentiation is observed in the structure analysis with K = 2 (**Figure 5**). Higher levels of K (with lower explanatory power for the distribution of genetic diversity according to H’ and ΔK values) partitions the genetic diversity of the wild accessions while the cultivated material remains a single cluster. The PCA analysis (**Figure 7**) separates the landrace Bredånger (L-2486) from the cultivars, but there is no differentiation among the Swedish and Norwegian cultivars. This is in agreement with previous studies of red clover where cultivars, landraces and breeding populations from the different Nordic countries were not clearly separated (Osterman et al., 2021).

In contrast, we can observe signs of geographic clustering among the wild populations where isolation-by-distance is suggested by the significant correlation between pairwise F_ST_ values and geographic distances. In the sample-level PCA analysis of only the wild accessions, the two wild Swedish accessions form a group separate from the Norwegian wild samples, albeit with W-1572 generation 1 and 2 overlapping along PC1 (**Figure 8**). A similar distinction was detected by Osterman et al. (2021) using different accessions from the same region. It should thus be possible to identify genetic differentiation among wild red clover at a finer scale than the one previously described from Asia and Europe, and among different parts of Europe (Jones et al., 2020). The individual-level distribution of genetic diversity (**Figure 6** and **Supplementary Figure S2**; Osterman et al., 2021), however, shows that neither samples nor accessions can be considered genetically homogenous but rather make up a continuum of genetic diversity.

### 4.3 No apparent short-term effect of *ex situ* conservation on the level of diversity

Evolutionary change occurs in all genetically diverse populations and plant populations in active conservation are no exception. *Ex situ* conservation often occurs at relatively small population sizes and hence *ex situ* conserved populations are expected to lose genetic diversity due to drift at a faster pace than natural populations, typically consisting of a higher number of individuals. Theory predicts that at least 40 individuals should be used for regeneration to assure conservation of alleles at a frequency of 10% or more and to conserve rarer alleles down to 5%, at least 100 individuals are needed (Crossa, 1989). For the accessions included in the current study, population sizes during regeneration have been between 50 and 100 individuals (Solberg et al., 2015) and should therefore be enough to conserve alleles above 5 - 10%. However, also with relatively high number of plants used for regeneration (120), bottleneck effects have been detected (Negri and Tiranti, 2010) and a general pattern of decreased genetic diversity has been observed in *ex situ* conserved plants, in particular outcrossing plants (Wei and Jiang, 2021). In this study, however, we find no support for either a systematic loss or gain of genetic diversity over the studied generations. Samples from later generations have neither more nor less genetic diversity than the original collections or the *in situ* recollection samples (**Figure 3**). Hence, with regards to conserving the level of genetic diversity we observe no difference between the *ex situ* and *in situ* conservation of Nordic red clover.

### 4.4 Changes in genetic composition during conservation

Previous morphological studies indicate significant directional changes during *ex situ* conservation in at least two of the eight wild Norwegian accessions studied here, and a general difference among generations for some morphological traits (Solberg et al., 2015). The observed change in these wild accessions was towards a morphology more typical for cultivars. In contrast with the morphological traits studied in Solberg et al. (2015) the microsatellite markers used in the current study are presumably selectively neutral. Pairwise F_ST_ values are in general low and non-significant within accessions (**Supplementary Table S6**) and no overall differences are detected between comparisons of original samples, different generations and the recollections (**Figure 4**). However, a more detailed picture emerges from the genetic clustering analyses and investigation of individual accessions.

For the accession W-1572 the original collect and the recollect are located relatively close to each other in the PCA but separate along PC2 (**Figure 8**). A large differentiation between the two might be expected since the meadow where the original sample was collected is now gone and replaced by a road and buildings, and the recollection was made on a roadside nearby. However, the pairwise F_ST_ value between the original collect and the recollect is lower than comparisons involving regeneration samples (**Supplementary Table S6**). The *STRUCTURE* analysis similarly shows a change in the genetic composition during *ex situ* conservation through an increase of the yellow component in generation 1 and 2 (**Figure 6**). This could suggest genetic drift during *ex situ* conservation, causing an increase in the frequency of a certain genotype (the yellow one), but the genetic diversity of the regeneration samples (0.732 for generation 1 and 0.725 for generation 2, respectively) is not markedly lower than that of the original collect. Solberg et al. (2015) also identify this accession to have changed significantly in some morphological traits from the original collect to generation 1 and suggest either gene flow or selection as probable causes. Whatever the cause, we observe larger changes *ex situ* than in the plants remaining in the wild.

In contrast, in W-1577 the data suggest higher differentiation between the original sample and the recollect than between the original and the regenerated sample. Pairwise F_ST_ values are higher when involving the recollect sample than when not and in the PCA the recollect sample is separated from the other two samples along PC2 (**Figure 7**). The old meadow where the original sample was collected is today replaced by cultivated land and the recollected sample was taken from a small semi-wild area between two copses of trees within a cultivated landscape. As this population is unprotected in the wild, it is unlikely to survive for long. It is fortunate that the *ex situ* conservation in the gene bank has conserved the variation in the original sample, even though the original population is most likely is the result of gene flow from a cultivar as discussed above.

The accession W-1571 is a particular case. Although the levels of genetic diversity of the different samples are comparable, it is the only accession with significant within-accession F_ST_ values. In both cases these F_ST_ values involved the sample from generation 1, W-1571-gen1. The differentiation of W-1571-gen1 from the original collect can also be seen in the *STRUCTURE* analysis (**Figure 6**) and for morphological traits (Solberg et al., 2015). The reason for this discrepancy is not clear, although a mislabeling is possible. Regardless, it seems clear that the correct genetic identity has been restored by generation 2.

Based on the genetic changes detected during *in* and *ex situ* conservation in this study we conclude that neither conservation method can be considered consistently superior to the other within the studied time frame and with neutral genetic markers. Chance events and the relative balance of evolutionary forces in any given population will determine the degree to which the genetic integrity of an accession is conserved. To some extent these forces can, and should, be controlled and minimized in *ex situ* conservation (FAO, 2014; AEGIS, 2023), but they will always be present.

Red clover is not a protected species in Norway or Sweden and none of the wild populations are located within protected areas. For some of the populations from which collections were made in 1980, the original location where the sample was collected no longer contain red clover. Among the eight Norwegian accessions three were originally sampled in areas that are today replaced by urban development or cultivated fields. This highlights the importance of *ex situ* conservation of genetic resources and stresses the need for active *in situ* conservation to assure long-term survival and continued evolution.

## Data availability statement

The original contributions presented in the study are included in the article/**Supplementary Material**. Further inquiries can be directed to the corresponding author.

## Author contributions

JH, PM, LO, SS, AA and AP contributed to conception and design of the study. PM, LO and AP carried out field collections. KA carried out the lab work. JH carried out the genetic analysis. JH and AP wrote the first draft of the manuscript with help from KA. All authors contributed to the article and approved the submitted version.
