# Supplementary material for: Limited genetic changes observed during in situ and ex situ conservation in Nordic populations of red clover (Trifolium pratense)
Source: Front Plant Sci. 2023 Aug 9;14:1233838. doi: 10.3389/fpls.2023.1233838 (PMC10445542; doi:10.3389/fpls.2023.1233838)
Supplement: Supplementary file 7 [file DataSheet_1.pdf]

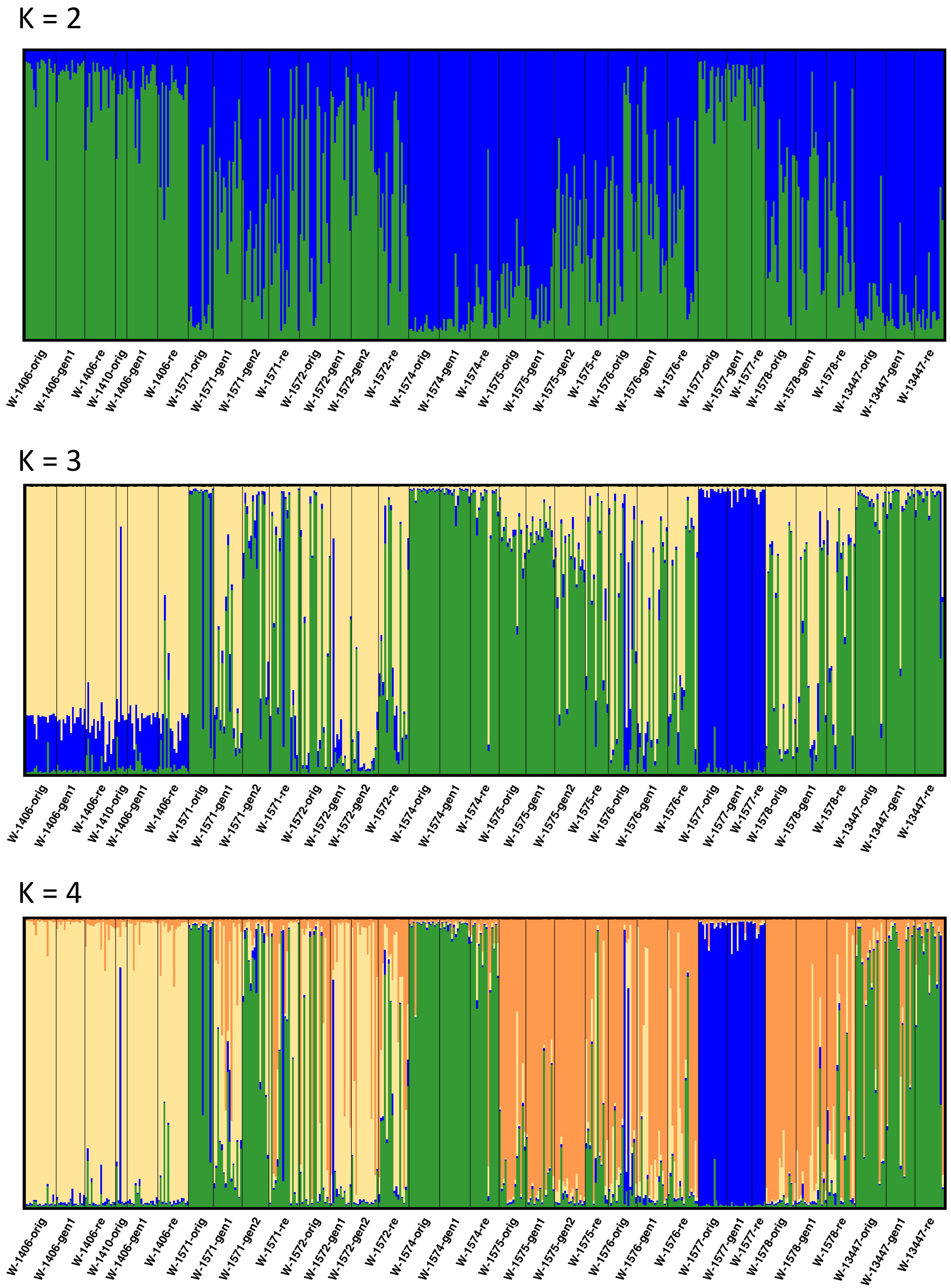

Supplementary Figure S1. Results of STRUCTURE analysis excluding cultivars and landrace. K = 2 through 4
