# Supplementary material for: Limited genetic changes observed during in situ and ex situ conservation in Nordic populations of red clover (Trifolium pratense)
Source: Front Plant Sci. 2023 Aug 9;14:1233838. doi: 10.3389/fpls.2023.1233838 (PMC10445542; doi:10.3389/fpls.2023.1233838)
Supplement: Supplementary file 8 [file DataSheet_2.pdf]

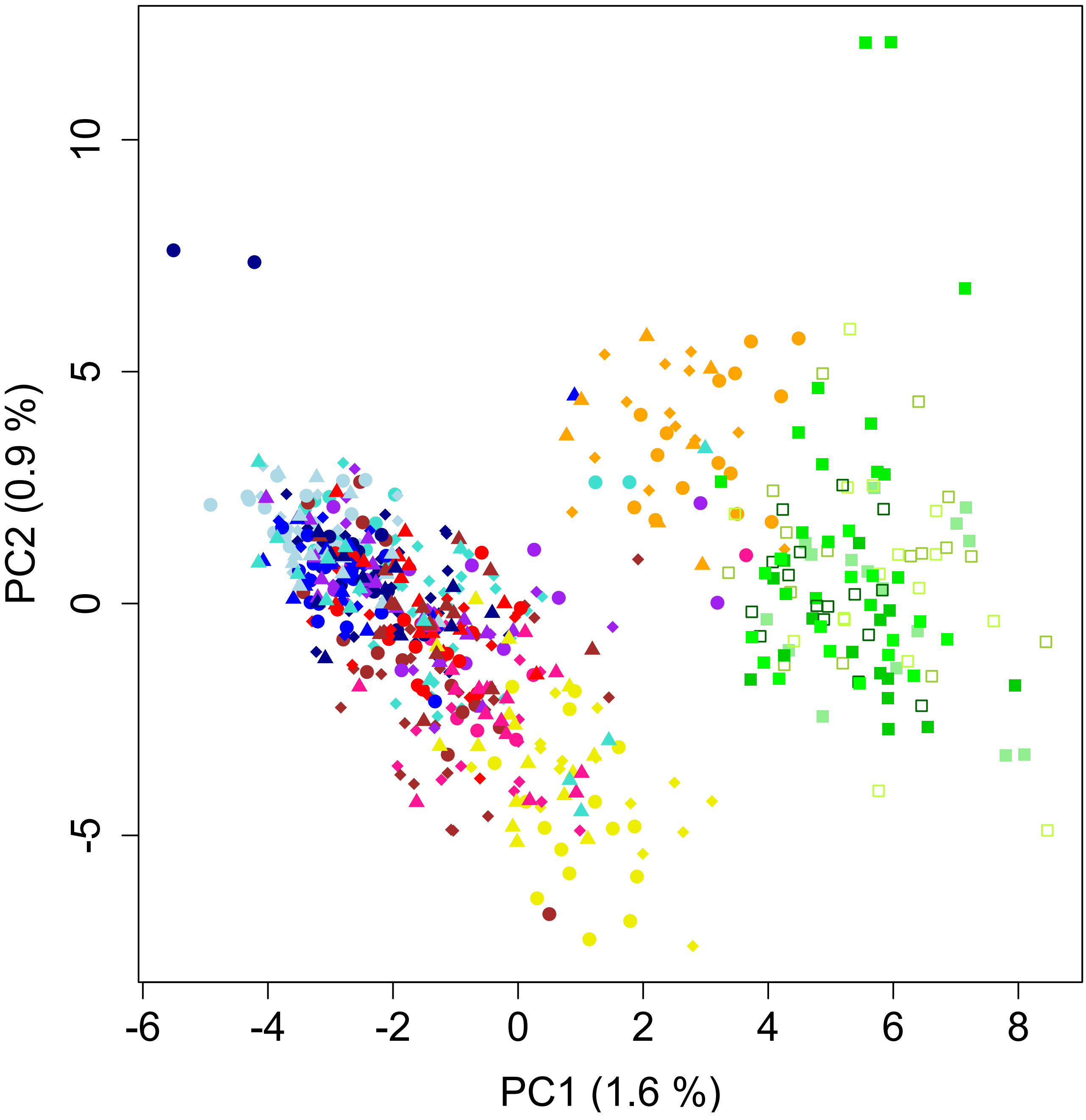

Supplementary Figure S2. Results of PCA based on individual genotypes. Colours and shapes as in Figure 7.
